# What Do We Know about Antimicrobial Activity of Astaxanthin and Fucoxanthin?

**DOI:** 10.3390/md20010036

**Published:** 2021-12-29

**Authors:** Tomasz M. Karpiński, Marcin Ożarowski, Rahat Alam, Małgorzata Łochyńska, Mark Stasiewicz

**Affiliations:** 1 Chair and Department of Medical Microbiology, Poznań University of Medical Sciences, Wieniawskiego 3, 61-712 Poznań, Poland; 2 Department of Biotechnology, Institute of Natural Fibres and Medicinal Plants, Wojska Polskiego 71b, 60-630 Poznań, Poland; marcin.ozarowski@iwnirz.pl (M.O.); malgorzata.lochynska@iwnirz.pl (M.Ł.); 3 Department of Genetic Engineering and Biotechnology, Faculty of Biological Science and Technology, Jashore University of Science and Technology, Jashore 7408, Bangladesh; rahat_160619@just.edu.bd; 4 Biological Solution Centre (BioSol Centre), Farmgate, Dhaka 1215, Bangladesh; 5 Research Group of Medical Microbiology, Chair and Department of Medical Microbiology, Poznań University of Medical Sciences, Wieniawskiego 3, 61-712 Poznań, Poland; 84682@student.ump.edu.pl

**Keywords:** mechanisms of action, efflux pump, biofilm inhibition, toxicity, safety, carcinogenicity

## Abstract

Astaxanthin (AST) and fucoxanthin (FUC) are natural xanthophylls, having multidirectional activity, including antioxidant, anti-inflammatory, and anticancer. Both compounds also show antimicrobial activity, which is presented in this review article. There are few papers that have presented the antimicrobial activity of AST. Obtained antimicrobial concentrations of AST (200–4000 µg/mL) are much higher than recommended by the European Food Safety Authority for consumption (2 mg daily). Therefore, we suggest that AST is unlikely to be of use in the clinical treatment of infections. Our knowledge about the antimicrobial activity of FUC is better and this compound acts against many bacteria already in low concentrations 10–250 µg/mL. Toxicological studies on animals present the safety of FUC application in doses 200 mg/kg body weight and higher. Taking available research into consideration, a clinical application of FUC as the antimicrobial substance is real and can be successful. However, this aspect requires further investigation. In this review, we also present potential mechanisms of antibacterial activity of carotenoids, to which AST and FUC belong.

## 1. Introduction

**Figure 1:**
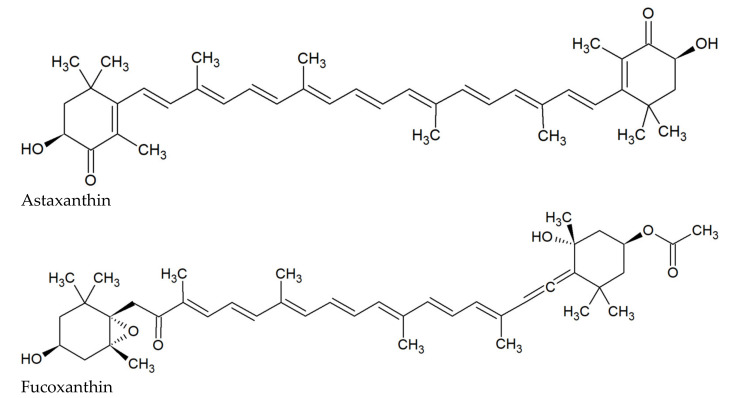
Chemical structures of AST and FUC.

Astaxanthin (AST) and fucoxanthin (FUC) are natural xanthophyll pigments belonging to carotenoids [1]. AST was first isolated from lobster by Kuhn and Sorensen in 1938 [2]. The AST is 3,3′-dihydroxy-β,β-carotene-4,4′-dione (Figure 1), and its molecular formula is C_40_H_52_0_4_ [3]. It was demonstrated that AST is produced by some microorganisms, including bacteria (e.g., *Escherichia coli*, *Mycobacterium lacticola, Paracoccus carotinifaciens*, and *Brevibacterium* sp.), green microalgae (e.g., *Haematococcus pluvialis, Chlorella zofingiensis*, and *Chlamydomonas nivalis*) and yeasts (e.g., *Phaffia rhodozyma* = *Xanthophyllomyces dendrorhous*, and *Rhodosporidium toruloides*) [4,5,6].

FUC was first isolated from the seaweeds *Fucus*, *Dictyota*, and *Laminaria* by Willstätter and Page in 1914 [7]. The FUC is 3′-acetoxy-5,6-epoxy-3,5′-dihydroxy-6′,7′-didehydro-5,6,7,8,5′,6′-hexahydro-β,β-carotene-8-one (Figure 1), and its molecular formula is C_42_H_58_O_6_ [8]. FUC is present in brown seaweeds belonging among others to the genera *Dictyota*, *Ecklonia*, *Fucus*, *Hijikia*, *Laminaria*, *Petalonia*, *Sargassum*, *Scytosiphon*, *Sphaerotrichia*, and *Undaria* [7,8,9,10,11,12].

Many studies showed that AST and FUC have antioxidant [12,13,14,15,16], anti-inflammatory [14,17,18,19,20], and anticancer [21,22,23,24,25] activities. It was also demonstrated that these phycochemical constituents have anti-diabetic [26,27], anti-obesity [28,29,30], and neuroprotective effects [31,32,33]. Both compounds also contain antimicrobial activity, and this aspect is presented in the review.

Therefore, the aim of this review is to show the recent data concerning the antibacterial, antifungal, antiviral, and antiparasitic activity of AST and FUC. The PubMed, Medline, and Scopus databases were used to search for articles using the keywords “astaxanthin”, “fucoxanthin”, “antibacterial”, “antifungal”, “antiviral”, “antiparasitic”, “activity”, and “effect”. Additionally, a manual review of references from the obtained literature was performed. Papers in which applied doses/concentrations of AST or FUC were missing were excluded from this review.

## 2. Astaxanthin

There is a small number of studies presenting the antimicrobial activity of AST. Shanmugapriya et al. [34] demonstrated that AST in the form of nanoemulsion can act against various bacteria. MIC values for Gram-positive and Gram-negative species were 500–4000 µg/mL [34]. In the in vitro study of *Trypanosoma cruzi*, the viability of the parasite was decreased in doses of AST 200-300 µg/mL. Simultaneously, the authors did not observe the therapeutic effect of AST against acute *T. cruzi* infection in the mice model [35]. In animal studies of mice infected with *Helicobacter pylori*, it was shown that AST has an impact on the decrease of bacterial abundance in the stomach. AST also had an anti-inflammatory effect reducing gastric inflammation and cytokine production by splenocytes [36]. In addition, some studies have shown a significant decrease of gastric *H. pylori* colonization in mice treated with an algal meal rich in AST [37]. In in silico studies, it was demonstrated that some xanthophylls, including AST, can inhibit SARS-CoV-2, acting on viral papain-like protease [38].

Recently, recommended or approved doses of AST in different countries amount to between 2 and 24 mg. According to the European Food Safety Authority, the proposed acceptable daily intake of AST is 2 mg [39]. AST is approved by the United States Food and Drug Administration (FDA) as generally recognized as safe (GRAS) for animals and humans for use in food [40,41]. It is important that according to the EU Regulation (EC) No. 1925/2006, the synthetic AST is not allowed for use in food, and that it has no GRAS status in the US [3,42]. Spiller and Dewell demonstrated that AST is safe in a dosage of 6 mg/day [40]. The EFSA Panel on Dietetic Products, Nutrition, and Allergies recommended that the daily dose of AST should not exceed 4 mg (0.06 mg/kg bw/day for a 70-kg person) [43]. Many studies have demonstrated that AST is also safe in much higher doses, up to 40 mg, without developing side effects [44,45,46]. Simultaneously, other papers demonstrated the following side effects: an increased frequency in bowel movement [47], stomach/abdominal pain [48], itch, dyspepsia, muscle pain, or diarrhea [49]. In animal studies, AST in repeat-doses of 100, 250, or 500 mg/kg bw showed no organ, hematological and biochemical abnormalities in pregnant mice [50]. In research with the use of Ames and in vitro micronucleus tests, a lack of AST genotoxicity has been shown. In the same paper, in a 2-year-long study on mice, the authors did not show the carcinogenicity potential of AST. However, in rats carcinogenicity of AST was observed in doses of 200 and 1000 mg/kg bw/day. Hepatocellular adenoma was developed in 18% of rats fed with 200 mg AST/kg bw/day and in 28% of animals fed with 1000 mg/kg bw/day [41].

The antimicrobial concentrations of AST, presented in Table 1, are much higher than recommended for consumption. This means that despite being active in high concentrations, AST is unlikely to be of clinical use in the treatment of infections or as an additional antimicrobial compound.

## 3. Fucoxanthin

Our knowledge about the antimicrobial activity of FUC is better than that of AST. In in vitro studies, FUC often acts against aerobic bacteria in low concentrations 10–250 µg/mL but has poor activity against anaerobic bacteria with MICs >1000 µg/mL [8,51]. In the big study, including 20 bacterial species, FUC acted against 13 aerobic bacteria. Obtained MICs for Gram-positive bacteria were between 62.5 and 250 µg/mL (median 125 µg/mL), while for Gram-negative ones were from 125 to 500 µg/mL (median 250 µg/mL) [8]. In another study including three Gram-positive and three Gram-negative bacteria, FUC extracted from *Turbinaria triquetra* had better activity, already in concentrations from 10–100 µg/mL [51]. Interestingly, very low MIC values were demonstrated against *Mycobacterium tuberculosis*: these were 2.8–4.1 µM (1.85–2.7 µg/mL) [52]. In a much higher concentration of 1000 µg/mL, FUC also acts against *Listeria monocytogenes* [53]. According to our previous studies, in the case of natural compounds, including flavonoids, organic acids, and curcumin, values of MIC above 1000 µg/mL should be considered as poor activity or lack of activity [54,55,56]. So high concentrations have no real therapeutic application and should be marked as inactive against microorganisms. A very high concentration of FUC, amounting to 4250 µg/mL, was used in the study of Liu et al. [57] against *S. aureus*, *Enterococcus* sp., *B. subtilis*, and *P. aeruginosa*. In another work by Peraman and Nachimuthu [58], MIC values of FUC were obtained against bacteria and also fungi (*Aspergillus brasiliensis*, *A. fumigatus* and *Candida albicans*), and amounted to 1000–4000 µg/mL.

In animal studies with chickens, it was observed that diet supplemented with 100 mg/kg or 200 mg/kg FUC meaningfully reduced the amount of Enterobacteriaceae, total mesophilic aerobic bacteria (TMAB), *Staphylococcus* spp., and *Pseudomonas* spp. from one to six days, in comparison to standard feed, without FUC. It is very interesting that FUC had low or no effect on the count of probiotic bacteria *Lactobacillus* spp. [59].

There are also individual studies concerning antiviral and antiparasitic effects of FUC. FUC from a brown alga *Dictyota* sp. acted against Herpes simplex virus type 1 and *Plasmodium falciparum* [60]. The activity of FUC against *Plasmodium falciparum* was also shown on a Chinese hamster ovarian cell line, with simultaneously relatively low cytotoxicity on the cell line [61].

There are no recommendations of acceptable daily intake of FUC; however, as shown by some studies, FUC is already active in low concentrations of 10–250 µg/mL.

Toxicological studies on rats demonstrated that a 28-day repeated oral dosing of fucoxanthin (95% purity) on rats in doses of 10 and 50 mg/kg/day did not show toxicity [62]. By contrast, oil containing FUC was safe in a dose of 200 mg/kg body weight in rats over a period of 13 weeks of feeding. Doses higher than 2000 mg/kg body weight led to 50% mortality [63]. Another study on mice confirmed the safety of single orally administered doses of 1000 and 2000 mg/kg and repeated doses of 500 and 1000 mg/kg administered for 30 days. In the study, no mortality or abnormalities were observed [64]. Both above studies indicate that FUC has a high level of safety. This means that a clinical application of FUC is more likely to be successful than AST, in addition to its use as an antimicrobial substance.

The antimicrobial activity of FUC is presented in Table 2.

## 4. Potential Mechanisms of Antibacterial Action of AST and FUC

**Figure 2:**
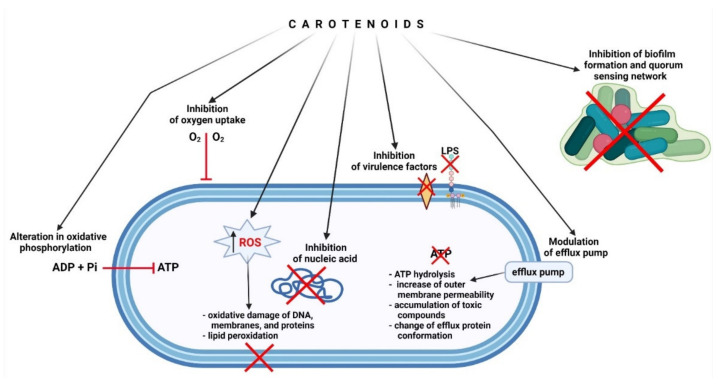
Potential antibacterial mechanisms of action of carotenoids, such as astaxanthin and fucoxanthin.

The mechanisms of the antimicrobial activity of xanthophylls are little known. However, it is very likely that potential mechanisms of action of xanthophylls are the same or similar to the mechanisms described in the carotenoids and terpenoids class, to which xanthophylls belong. It was recently discovered that small terpenoids, like carvacrol, can act directly on bacterial cell and membrane, leading to damage of the cell wall and membrane and leakage of cell content [65]. Terpenoids have also the ability to permeabilize and depolarize the cytoplasmic membrane [43]. It was also observed that FUC can increase cell membrane permeability and thus the leakage of cytoplasm [66]. AST and FUC are compounds, having 40 and 42 carbon atoms, respectively. Therefore, xanthophylls are more similar to antibiotic molecules, like macrolides, which also have about 40 carbon atoms. This suggests that their remaining mechanisms of action may require a link to a protein receptor or nucleic acid. Some data from the literature indicate that terpenoids, including carotenoids, can modulate efflux pumps [67,68,69]. The exact mechanism was described among others in alkaloids and flavonoids [70,71]. Moreover, terpenoids can lead to the accumulation of toxic compounds inside bacteria and can have an impact on ATP hydrolysis, leading to disturbance of efflux pump activation. In Gram-negative bacteria, these compounds can increase the permeability of the outer membrane and can change the conformation of efflux protein structures [67,68]. Terpenoids can also lead to the accumulation of intracellular reactive oxygen species (ROS), which can damage the bacterial cells, causing oxidative damage of membranes, DNA, proteins, and lipid peroxidation [72,73,74]. Another mechanism of action of terpenoids, including carotenoids and xanthophylls, is the inhibition of biofilm formation, both in bacteria and fungi. This effect can be triggered by inhibition of biofilm matrix formation, decreasing cell adhesion, inhibition of the virulence factors, e.g., toxin production, and blocking the quorum sensing network [75,76,77]. Action through inhibition of bacterial virulence and blocking of quorum sensing and biofilm was described in flavonoids [70]. Anti-quorum and anti-biofilm activities were also demonstrated for two xanthophylls: lutein and zeaxanthin [78,79]. Some studies confirmed that FUC can affect lipopolysaccharide (LPS), an endotoxin of Gram-negative bacteria, which impacts inflammatory response. FUC can suppress the NF-κB activation and inhibit the production of pro-inflammatory cytokines induced by LPS [80,81,82]. It was also suggested that FUC can act antibacterial by nucleic acid inhibition [66]. Mahizan et al. [67] proposed two more mechanisms: inhibition of oxygen uptake and alteration in oxidative phosphorylation. Terpenoids act mainly against aerobic bacteria, for which oxidative phosphorylation is a key biochemical process responsible for cellular respiration. The action of terpenoids leads to a reduction in oxygen concentration, disturbance of bacterial respiration, and ultimately death of the bacteria [83,84]. Potential antibacterial mechanisms of carotenoids, such as AST and FUC, are presented in Figure 2.

## 5. Conclusions

As demonstrated in the literature, antimicrobial concentrations of astaxanthin are much higher than recommended by the European Food Safety for consumption. Therefore, this compound is unlikely to be of clinical use in the treatment of infections. In the case of fucoxanthin, it acts against many bacteria in low concentrations. Simultaneously, toxicological studies present the safety of fucoxanthin application in high doses. Therefore, a clinical application of fucoxanthin as an antimicrobial substance is real and can be successful. However, this aspect requires further research.

## Figures and Tables

**Table 1:** Antimicrobial activity of astaxanthin.

Targeted Microorganism	Antimicrobial Doses	References
*Bacillus subtilis*	in vitro, nanoemulsion, MIC 500–4000 µg/mL	[34]
*Escherichia coli*	in vitro, nanoemulsion, MIC 500–4000 µg/mL	[34]
*Helicobacter pylori*	in vivo, mice, 200 mg per kg body weight per day	[36]
*Pseudomonas aeruginosa*	in vitro, nanoemulsion, MIC 500–4000 µg/mL	[34]
*Staphylococcus aureus*	in vitro, nanoemulsion, MIC 1000–2000 µg/mL	[34]
*Streptococcus mutans*	in vitro, nanoemulsion, MIC 500–2000 µg/mL	[34]
SARS-CoV-2	in silico	[38]
*Trypanosoma cruzi*	in vitro, 200-300 µg/mL; lack of in vivo activity	[35]

**Table 2:** Antimicrobial activity of fucoxanthin.

Targeted Microorganism	Antimicrobial Doses	References
*Acinetobacter lwoffi* *i*	in vitro, MIC 250 µg/mL	[8]
*Actinomyces israelii*	in vitro, MIC > 1000 µg/mL	[8]
*Atopobium parvulum*	in vitro, MIC > 1000 µg/mL	[8]
*Bacillus cereus*	in vitro, 10–100 µg/mL	[51]
*Bacillus subtilis*	in vitro, 10–100 µg/mL	[51]
	in vitro, MIC 4000 µg/mL	[58]
	in vitro, 4250 µg/mL	[57]
*Enterobacteriaceae*	in vivo, chicken, 100–200 mg/kg	[59]
*Enterococcus* sp.	in vitro, 4250 µg/mL	[57]
*Enterococcus faecalis*	in vitro, MIC 125–250 µg/mL	[8]
	in vitro, 4250 µg/mL	[57]
*Escherichia coli*	in vitro, 10–100 µg/mL	[51]
	in vitro, MIC 125 µg/mL	[8]
	in vitro, MIC 2000 µg/mL	[58]
*Klebsiella oxytoca*	in vitro, MIC 125–250 µg/mL	[8]
*Klebsiella pneumoniae*	in vitro, 10–100 µg/mL	[51]
	in vitro, MIC 250 µg/mL	[8]
	in vitro, MIC 1000 µg/mL	[58]
*Listeria monocytogenes*	in vitro, 1000 µg/mL	[53]
*Mitsuokella multacida*	in vitro, MIC > 1000 µg/mL	[8]
*Mycobacterium tuberculosis*	in vitro, MIC 1.85–2.7 µg/mL	[52]
*Peptococcus niger*	in vitro, MIC > 1000 µg/mL	[8]
*Porphyromonas gingivalis*	in vitro, MIC > 1000 µg/mL	[8]
*Propionibacterium acnes*	in vitro, MIC > 1000 µg/mL	[8]
*Proteus mirabilis*	in vitro, MIC 500 µg/mL	[8]
*Pseudomonas* spp.	in vivo, chicken, 100–200 mg/kg	[59]
*Pseudomonas aeruginosa*	in vitro, 10–100 µg/mL	[51]
	in vitro, MIC 250–500 µg/mL	[8]
	in vitro, MIC 1000 µg/mL	[58]
	in vitro, 4250 µg/mL	[57]
*Staphylococcus* spp.	in vivo, chicken, 100–200 mg/kg	[59]
*Staphylococcus aureus*	in vitro, 10–100 µg/mL	[51]
	in vitro, MIC 125 µg/mL	[8]
	in vitro, MIC 1000 µg/mL	[58]
	in vitro, 4250 µg/mL	[57]
*Staphylococcus epidermidis*	in vitro, MIC 125 µg/mL	[8]
*Streptococcus agalactiae*	in vitro, MIC 62.5 µg/mL	[8]
*Streptococcus pneumoniae*	in vitro, MIC 125 µg/mL	[8]
*Streptococcus pyogenes*	in vitro, MIC 125 µg/mL	[8]
*Serratia marcescens*	in vitro, MIC 500 µg/mL	[8]
*Veilonella parvula*	in vitro, MIC > 1000 µg/mL	[8]
Total mesophilic aerobic bacteria (TMAB)	in vivo, chicken, 100–200 mg/kg	[59]
*Aspergillus brasiliensis*	in vitro, MIC 2000 µg/mL	[58]
*Aspergillus fumigatus*	in vitro, MIC 1000 µg/mL	[58]
*Candida albicans*	in vitro, MIC 2000 µg/mL	[58]
Herpes simplex virus type 1	in vitro, IC50 5 µg/mL	[60]
*Plasmodium falciparum*	in vitro, EC50 2.9 µg/mL	[60]
	in vitro, IC50 1.3 µg/mL (1.5 µM)	[61]
